# Amotivation and Academic Engagement in Western Romanian University Students: A Conditional Self-Regulation Model with Forethought and Self-Reflection Under Perceived Performance Control

**DOI:** 10.3390/brainsci16030313

**Published:** 2026-03-15

**Authors:** Alina Roman, Horațiu Catalano, Karla Barth, Cristina Florescu, Mariana Tipei-Voia, Dana Rad, Olga Chiș, Edgar Demeter, Regis Roman, Raluca Șandru, Irina Mihaela Trifan

**Affiliations:** 1 Faculty of Psychology and Educational Sciences, Babeş-Bolyai University of Cluj-Napoca, 400029 Cluj-Napoca, Romania; romanalinafelicia@yahoo.com (A.R.); horatiu.catalano@ubbcluj.ro (H.C.); mariana.cismasiu@ubbcluj.ro (M.T.-V.); olga.chis@ubbcluj.ro (O.C.); 2 Centre of Research Development and Innovation in Psychology, Faculty of Educational Sciences, Aurel Vlaicu University of Arad, 310032 Arad, Romania; edgar.demeter@uav.ro; 3 Faculty of Socio-Humanistic Sciences, University of Oradea, 410087 Oradea, Romania; karla_barth@yahoo.com (K.B.); mariacristinaflorescu@yahoo.com (C.F.); 4 Faculty of Humanities and Social Sciences, Aurel Vlaicu University of Arad, 310032 Arad, Romania; regis_roman@yahoo.com; 5 Business Faculty, Babeş-Bolyai University of Cluj-Napoca, 400084 Cluj-Napoca, Romania; raluca.sandru@ubbcluj.ro; 6 Pharmacy, Science, and Technology of Târgu Mureș, George Emil Palade University of Medicine, 540142 Târgu Mureș, Romania; irina.trifan@umfst.ro

**Keywords:** self-regulation, academic adaptation, amotivation, academic engagement, perceived performance control, conditional effects, university students

## Abstract

**Highlights:**

**What are the main findings?**

- Among Western Romanian university students, amotivation showed a robust direct association with academic engagement across low, mean, and high levels of perceived performance control (attenuated, but not eliminated, as control increased).
- Self-regulation pathways were process-specific and context-dependent: perceived performance control moderated the amotivation → forethought link (stronger under low control), whereas self-reflection mediated the amotivation–engagement link only at moderate levels of control.

**What are the implications of the main findings?**

- Findings support a ‘controlled direct association’ architecture, indicating that the amotivation–engagement link persists across low, mean, and high perceived performance control levels, even as its magnitude decreases.
- Interventions should prioritize reducing amotivation and optimizing performance control, while applying self-regulation training selectively (e.g., strengthening reflective regulation in moderate-control contexts rather than assuming planning skills universally translate into engagement).

**Abstract:**

Background/Objectives: Academic engagement plays a central role in students’ learning outcomes and persistence in higher education. However, the mechanisms through which amotivation influences engagement remain insufficiently understood, particularly within conditional self-regulation frameworks. The present study examined a conditional self-regulation model in which amotivation predicts academic engagement through forethought and self-reflection under different levels of perceived performance control. Methods: Data were collected from 530 university students from Western Romania. A moderated parallel mediation model (PROCESS Model 59) was estimated to test whether forethought and self-reflection mediate the relationship between amotivation and academic engagement and whether perceived performance control moderates these pathways. Results: The results indicated that amotivation maintained a robust direct association with academic engagement across levels of performance control. Perceived performance control moderated the amotivation–forethought pathway, while self-reflection showed conditional indirect effects depending on control levels. Conclusions: These findings suggest that motivational deficits operate within a context-sensitive regulatory architecture in which control beliefs shape the activation of self-regulatory processes. The results contribute to understanding academic adaptation under motivational constraints and highlight the role of perceived performance control in students’ self-regulation systems.

## 1. Introduction

The transition to university represents a critical developmental period characterized by increased cognitive demands, heightened performance evaluation, and greater responsibility for self-directed learning [1,2,3,4,5,6,7]. During this phase, students are required not only to acquire new academic knowledge but also to adapt to unfamiliar learning environments, regulate their motivation, and sustain engagement under conditions of uncertainty and evaluative pressure [5,7]. Difficulties in academic adaptation during this transition have been consistently associated with reduced academic engagement, lower performance, and increased dropout intentions, underscoring adaptation as a central mechanism shaping students’ academic trajectories [8]. Research on student adjustment indicates that socio-cultural and academic adaptation processes play a central role in shaping students’ satisfaction and integration within the university environment [1]. Empirical studies on international and domestic students have shown that challenges related to academic expectations, learning strategies, and institutional demands can significantly affect students’ ability to adapt to higher education contexts [1,2,3].

Recent systematic reviews further emphasize that adaptation difficulties are particularly pronounced among first-generation students and other vulnerable groups, often leading to disparities in academic achievement and persistence [2,3]. Institutional support mechanisms and transition programs have therefore been identified as critical factors facilitating students’ successful adjustment to university life [4,5]. Contemporary research also highlights that the initial phase of university studies represents a sensitive developmental period in which academic and social integration processes strongly influence students’ trajectories [4,5].

In this context, adaptation is closely connected to students’ academic self-efficacy and expectations, which shape how they respond to academic demands and learning challenges [6,7,8]. Interventions aimed at strengthening motivation and self-regulation have been shown to improve students’ capacity to navigate this transition successfully [7,8]. Conversely, uncertainty and difficulties in adapting to academic requirements may increase the likelihood of dropout decisions during the early stages of university education [8]. These dynamics are closely linked to academic engagement, a multidimensional construct reflecting students’ behavioral, cognitive, and emotional investment in learning activities [9,10]. Empirical studies have demonstrated that lower levels of engagement are associated with stronger dropout intentions and weaker academic persistence [10]. At the same time, engagement has been identified as a key psychological resource supporting academic performance and long-term educational success among university students [10,11].

Academic engagement is widely recognized as a central predictor of learning outcomes in higher education, reflecting students’ active involvement in academic activities and their commitment to learning goals [12]. Empirical evidence indicates that higher levels of engagement are consistently associated with improved academic performance and stronger learning achievements among university students [12]. In addition to its impact on performance, academic engagement is also strongly related to persistence in higher education and a reduced likelihood of dropout, making it a key factor in students’ academic adaptation and long-term educational trajectories [13]. Systematic reviews examining engagement across different educational contexts further highlight that students who remain actively involved in learning activities are more likely to sustain motivation and continue their studies despite academic challenges [13].

Recent research conceptualizes academic engagement as a multidimensional construct encompassing behavioral participation in learning tasks, cognitive investment in understanding course material, and emotional involvement in academic experiences [14]. Within this framework, engagement reflects not only observable study behaviors but also deeper psychological processes related to interest, effort, and perceived relevance of learning activities [14]. Such a multidimensional perspective has become increasingly prominent in contemporary higher education research, where engagement is viewed as a central mechanism linking students’ motivational resources with their academic success [14]. However, existing models often treat motivation, self-regulation, and engagement as additive predictors and rarely explain when self-regulatory processes transmit motivational deficits to engagement under different contextual conditions. This gap is particularly relevant during academic transition, when perceived performance control may shape whether students mobilize regulation or disengage.

A further unresolved issue concerns what may be termed an SRL paradox. Standard self-regulated learning frameworks generally assume that planning-oriented regulation is adaptively beneficial, as strategic planning and goal setting are typically associated with improved academic adjustment and learning engagement [6,13]. However, under conditions of low perceived control, regulatory activation may reflect compensatory effort rather than effective self-regulation [7]. In such situations, students may activate planning strategies without these efforts translating into sustained academic engagement or improved academic outcomes [10]. This points to a broader conditionality gap in the literature: the field still lacks a clear account of when self-regulatory processes fail to offset motivational depletion and under what contextual conditions their adaptive value is weakened, neutralized, or enabled within higher education contexts [3].

To address this gap, the present study tests a conditional self-regulation framework in which amotivation (X) predicts academic engagement (Y) directly and indirectly via forethought (M1) and self-reflection (M2), contingent on perceived performance control (W). The proposed model is examined using a moderated parallel mediation framework implemented with PROCESS Model 59.

Based on this framework, the present study addresses the following research questions:

RQ1. How does amotivation relate to academic engagement as a core indicator of academic adaptation among university students?

RQ2. Do self-regulatory processes—specifically forethought and self-reflection—transmit the effects of amotivation on academic engagement?

RQ3. Does perceived performance control moderate the relationship between amotivation and self-regulatory processes involved in academic adaptation?

RQ4. Under what levels of perceived performance control do self-regulatory processes contribute to academic engagement?

RQ5. To what extent does perceived performance control attenuate—or fail to attenuate—the direct association between amotivation and academic engagement?

**Figure 1:**
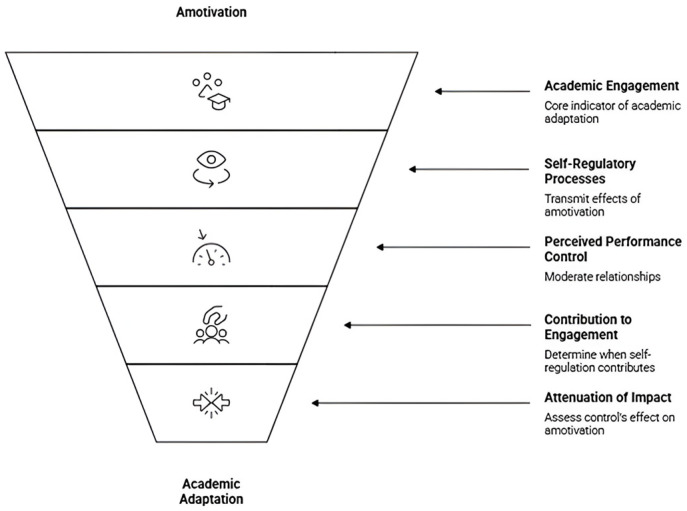
Conceptual model of the conditional self-regulation framework of academic adaptation.

As illustrated in Figure 1, the study proposes a conditional self-regulation model in which amotivation (X) predicts academic engagement (Y) both directly and indirectly through two parallel self-regulatory mechanisms: forethought (M1) and self-reflection (M2). Perceived performance control (W) is modeled as a contextual moderator that conditions the strength of the a-paths from amotivation to each mediator (X → M1; X → M2), consistent with a moderated parallel mediation framework (PROCESS Model 59). This specification allows the estimation of (i) the direct effect of amotivation on engagement (c′), (ii) the indirect effects via forethought and self-reflection, and (iii) conditional (moderated) direct and indirect effects across low, mean, and high levels of perceived performance control.

Building on the conditional self-regulation framework (Figure 1) and the moderated parallel mediation approach, we formulated the following hypotheses:

**H1.**  *Amotivation will be significantly associated with academic engagement.*

**H2.**  *The association between amotivation and academic engagement will be indirectly transmitted through self-regulatory processes, specifically forethought and self-reflection (parallel mediation).*

**H3.**  *Perceived performance control will moderate the effect of amotivation on self-regulatory processes (amotivation → forethought; amotivation → self-reflection), such that these links vary as a function of control.*

**H4.**  *The conditional indirect effects of amotivation on academic engagement through forethought and self-reflection will differ across levels of perceived performance control (moderated mediation).*

## 2. Literature Review

### 2.1. Academic Adaptation

Academic adaptation is widely conceptualized as a multidimensional process reflecting students’ capacity to manage academic demands, cope with learning-related stressors, and function effectively within the institutional context of higher education [4,5]. Empirical research across diverse educational systems indicates that successful adaptation is closely linked to academic engagement, which captures students’ sustained behavioral, cognitive, and emotional involvement in learning activities [7,8,9].

Academic engagement has emerged as a robust predictor of academic performance, persistence, and retention, functioning as a proximal indicator of students’ adaptive functioning within the university environment [10,11,12,13,14]. Engaged students demonstrate greater resilience, more effective coping strategies, and higher levels of academic achievement, whereas disengagement has been associated with burnout, withdrawal behaviors, and diminished well-being [15,16,17,18]. From this perspective, engagement is not merely an outcome of academic adaptation but a core regulatory state through which motivational and cognitive processes exert their influence.

### 2.2. Motivation Theories and Amotivation

Motivational resources play a foundational role in academic adaptation by energizing goal-directed behavior and sustaining effort over time. A substantial body of research has demonstrated that students’ motivational resources are positively associated with engagement, achievement, and persistence in higher education [19,20,21,22,23]. However, far less attention has been devoted to amotivation, a state characterized by diminished intentionality, perceived competence, and sense of purpose, despite evidence linking it to disengagement and dropout intentions [3,18].

Amotivation is particularly salient during the university transition, a context marked by ambiguous expectations, increased autonomy, and intensified performance pressure [4,22,23]. Under such conditions, students experiencing amotivation may struggle to initiate or sustain engagement even when cognitive abilities and external supports are available. This suggests that academic adaptation may be compromised not merely by insufficient resources but by the erosion of motivational foundations necessary for regulatory processes to function effectively.

### 2.3. Self-Regulation

Self-regulated learning frameworks emphasize the role of cognitive and metacognitive processes—such as forethought, planning, monitoring, and self-reflection—in supporting adaptive learning behavior [24,25,26]. Forethought processes facilitate goal setting and strategic orientation, whereas self-reflection enables evaluation and adjustment of learning strategies. Empirical evidence indicates that these processes are associated with higher engagement and academic success, particularly in challenging learning environments [15,27,28,29].

At the same time, emerging research suggests that self-regulation does not operate uniformly across contexts. Under sustained uncertainty, stress, or motivational depletion, regulatory resources may become fragile, fatigued, or insufficient to counteract disengagement [17,25,28]. Research on performance pressure further indicates that regulatory processes are sensitive to contextual appraisals of control, which may determine whether self-regulation functions adaptively or fails to prevent disengagement [23]. These findings challenge the assumption that self-regulation can consistently compensate for motivational deficits and highlight the need to examine the conditions under which regulatory processes remain effective.

### 2.4. Motivation–Self-Regulation Interaction and the Need for Conditional Modeling

Perceived performance control refers to students’ beliefs regarding their capacity to influence academic outcomes through effort and strategy. Higher levels of perceived control have been associated with greater persistence, proactive coping, and engagement, particularly in demanding academic contexts [30,31]. At a broader level, institutional and social support structures have been shown to influence academic performance indirectly through academic adaptation and engagement [14,32,33].

However, only limited research has examined the combined role of motivational deficits and self-regulation processes in explaining academic engagement in higher education contexts [7,10]. In particular, it remains unclear whether self-regulation can offset the association of amotivation across different control contexts or whether perceived control functions as a regulatory gate that enables, constrains, or reshapes adaptive responses. Addressing this gap is essential for understanding why some students remain engaged under motivational strain, whereas others disengage despite comparable resources.

Although prior research has examined the roles of motivation, self-regulation, and contextual support in academic adaptation, these factors are often investigated separately rather than as components of an integrated regulatory system [17]. Many existing studies rely primarily on direct-effect or simple mediation frameworks when explaining students’ academic engagement and adaptation processes [22]. Within these approaches, motivational deficits are frequently assumed to influence academic outcomes through relatively linear pathways involving self-regulatory mechanisms [22]. At the same time, regulatory processes are often conceptualized as functioning independently of contextual appraisals, such as students’ perceptions of control over academic performance or learning conditions [23]. However, both theoretical developments and recent empirical findings increasingly suggest that self-regulation is not an unconditional mechanism and does not operate uniformly across all learning contexts [23]. Instead, its effectiveness appears to depend on motivational resources, perceived control, and the broader academic environment in which regulation is activated [17].

In the context of academic adaptation, amotivation represents a critical destabilizing state that may be directly associated with engagement while simultaneously altering the functioning of regulatory processes. At the same time, perceived performance control may condition whether students mobilize regulatory strategies or disengage from academic demands. Capturing these dynamics requires an analytical approach capable of modeling both indirect pathways through self-regulation and conditional effects that vary as a function of perceived control.

To address this complexity, the present study employs a moderated parallel mediation framework, implemented through PROCESS Model 59 [10,34]. This model is particularly suited to the study’s theoretical objectives for three reasons. First, it allows for the simultaneous examination of multiple self-regulatory processes—forethought and self-reflection—thereby acknowledging the multidimensional nature of self-regulation. Second, it enables the testing of moderation at both the motivational-to-regulatory stage and the regulatory-to-engagement stage, aligning with theoretical perspectives that conceptualize perceived control as a contextual regulator rather than a simple additive resource. Third, Model 59 permits the estimation of conditional direct effects, making it possible to determine whether amotivation exerts a persistent influence on academic engagement even when regulatory processes and perceived control are taken into account. By adopting this analytical strategy, the study moves beyond linear mediation assumptions and examines academic adaptation as a control-gated regulatory system, in which motivation, self-regulation, and perceived performance control interact dynamically.

More specifically, the present study addresses a conditionality problem that remains insufficiently specified in prior self-regulated learning research. While regulatory processes such as forethought and self-reflection are typically conceptualized as adaptive resources, their effectiveness may depend on whether students perceive academic outcomes as controllable. This means that self-regulation should not be assumed to function uniformly across motivational contexts. Instead, the present model examines when self-regulatory processes fail to counteract motivational depletion and whether perceived performance control acts as the contextual condition that enables, constrains, or neutralizes their contribution to academic engagement.

## 3. Materials and Methods

The present study adopted a quantitative, cross-sectional research design to examine the conditional mechanisms through which amotivation is associated with academic engagement during the transition to university. Grounded in theoretical perspectives on self-regulated learning and academic adaptation, the study sought to determine whether the influence of amotivation on academic engagement operates directly or is transmitted through distinct self-regulatory processes; namely, forethought and self-reflection. In addition, the study explored whether these relationships vary as a function of students’ perceived performance control. To adequately reflect the multidimensional and context-sensitive nature of academic adaptation, a moderated parallel mediation framework was employed, enabling the simultaneous estimation of direct effects, indirect pathways through multiple regulatory processes, and conditional effects across levels of perceived control within a single analytical model [10].

### 3.1. Participants and Procedure

Participants were 530 university students enrolled in undergraduate, master’s, and doctoral programs at higher education institutions located in Western Romania. A convenience sampling strategy was employed, with participants recruited based on accessibility and voluntary participation during the academic year. This approach was considered appropriate given the exploratory aim of examining motivational and self-regulatory processes within a naturalistic academic context.

The sample consisted predominantly of female students (87.5%, *n* = 464), with male students representing 12.5% (*n* = 66). Participants’ ages ranged from 18 to 56 years (M = 28.86, SD = 9.75). With respect to educational background, 41.1% of respondents reported having completed secondary education, 38.9% held a bachelor’s degree, 15.5% a master’s degree, 0.8% reported doctoral studies, and 3.8% indicated other forms of education, including post-secondary vocational training. All participants were currently enrolled as university students at the time of data collection. The small “other” category (0.6%) reflects prior educational pathways before the current enrolment (e.g., a previous university degree/second-degree enrolment or other post-secondary qualifications), rather than a different current study status. Participants’ demographics are presented in Table 1.

Academic discipline and year of study were not collected in the present dataset; future studies should include these variables to enable discipline- and year-specific generalization.

Data were collected using an online self-report questionnaire administered during the academic year. Students were invited to participate through institutional communication channels and academic networks. Participation was entirely voluntary, and no incentives were provided. Prior to completing the survey, all participants were informed about the purpose of the study, the anonymous and confidential nature of the data collection, and their right to withdraw from the study at any point without consequences. Informed consent was obtained electronically from all respondents before participation.

### 3.2. Instruments

Amotivation was assessed using the amotivation subscale of the Academic Motivation Scale—College Version (AMS-C 28) developed by Vallerand et al. [22,35]. The subscale captures students’ lack of intentionality, diminished perceived competence, and reduced perceived value associated with academic activities. Items were rated on a 7-point Likert scale (1 = strongly disagree, 7 = strongly agree), with higher scores indicating higher levels of amotivation. In the present study, the amotivation subscale demonstrated high internal consistency (Cronbach’s α = 0.86), consistent with reliability coefficients reported in previous validation studies and in the authors’ earlier research using the same instrument.

Academic engagement was measured using a validated academic engagement scale assessing students’ vigor, persistence, and cognitive–emotional investment in learning activities [14,36]. The instrument conceptualizes engagement as a core indicator of academic adaptation. Items were rated on a 5-point Likert scale, with higher scores reflecting higher levels of engagement. In the present sample, the scale exhibited excellent internal consistency (Cronbach’s α = 0.94), in line with prior empirical evidence and the authors’ previous publications.

Self-regulatory processes were assessed using the Academic Self-Regulated Learning Questionnaire (ASLQ) developed by Nambiar et al. [37]. The ASLQ operationalizes self-regulation across three phases consistent with Zimmerman’s cyclical model—forethought, performance control, and self-reflection [37,38]. The forethought subscale assesses goal setting, planning, and anticipatory regulation of learning activities, whereas the self-reflection subscale captures evaluative and reflective processes related to learning outcomes and strategy use. The performance control dimension reflects students’ perceived capacity to influence academic outcomes through effort, strategy use, and in-task behavioral regulation (e.g., time management, managing distractions, and using supportive strategies during study) [37,38]. Items were rated on a 5-point Likert scale (1 = never true of me, 5 = always true of me), with higher scores indicating stronger engagement in the respective regulatory processes and stronger perceived performance control. An example item from the performance control dimension is: “I ask for help if I do not understand the study material.” In the present sample, the performance control subscale showed high internal consistency (Cronbach’s α = 0.95), comparable to reliability coefficients reported in prior work using the same measure.

### 3.3. Data Analysis Strategy

A conditional process approach was selected because the theoretical contribution of the study concerns when self-regulatory mechanisms transmit the association between amotivation and academic engagement under different levels of perceived performance control. Accordingly, we tested a moderated parallel mediation model (PROCESS Model 59), which simultaneously estimates (a) direct effects, (b) indirect effects through parallel mediators (forethought and self-reflection), and (c) conditional effects across levels of the moderator. This approach is appropriate for cross-sectional survey data, provides transparent estimation of conditional indirect effects using bootstrap confidence intervals, and allows probing moderation via Johnson–Neyman regions of significance. Although SEM is also suitable for mediation models, PROCESS was chosen here for its direct implementation of Model 59 and its standard reporting of conditional direct/indirect effects at theoretically meaningful levels of the moderator.

Data analyses were conducted using SPSS v26, with conditional process analyses performed using the PROCESS macro (version 4.0). To test the proposed conditional self-regulation framework, a moderated parallel mediation model corresponding to PROCESS Model 59 was estimated. Within this model, amotivation was specified as the independent variable (X), academic engagement as the outcome variable (Y), forethought (M1) and self-reflection (M2) as parallel mediating variables, and perceived performance control as the moderating variable (W).

Moderated parallel mediation was tested using the PROCESS macro for SPSS (Model 59), which enables estimation of conditional direct and indirect effects within an ordinary least squares regression framework, with effects evaluated at low (−1 SD), mean, and high (+1 SD) levels of the moderator [39,40]. To probe moderation effects, we applied the Johnson–Neyman technique to identify regions of significance for conditional effects across the observed range of perceived performance control [41,42]. Indirect effects were estimated using bootstrap confidence intervals (5000 resamples), consistent with current recommendations for conditional process models [39,40].

This analytical strategy was selected to allow for the simultaneous examination of direct, indirect, and conditional effects within a single coherent framework. Specifically, the model enabled the assessment of the direct association between amotivation and academic engagement, the extent to which this association is transmitted through distinct self-regulatory processes, and the degree to which perceived performance control conditions both motivational and regulatory pathways. By integrating mediation and moderation within the same analytical structure, this approach aligns with contemporary recommendations for examining complex psychological mechanisms and their contextual contingencies [40,41,42,43,44].

All continuous predictors were mean-centered prior to analysis to reduce multicollinearity and facilitate the interpretation of interaction effects. Indirect effects were estimated using a bootstrapping procedure with 5000 resamples, and 95% bias-corrected confidence intervals were computed. Conditional effects were further examined using the Johnson–Neyman technique to identify regions of significance across levels of perceived performance control. Statistical significance was evaluated using a threshold of *p* < 0.05.

Because standardized coefficients are not provided by PROCESS for models including moderator terms, we additionally estimated a complementary OLS outcome regression without interaction terms and report standardized coefficients (β) from that model to facilitate relative magnitude comparisons across predictors.

## 4. Results

Participants reported the following mean levels: academic engagement (M = 4.06, SD = 0.72), self-reflection (M = 4.06, SD = 0.72), forethought (M = 3.87, SD = 0.62), and perceived performance control (M = 3.76, SD = 0.54). Amotivation had a mean of M = 5.78 (SD = 1.34) on its scale. Variability across measures supported subsequent conditional process analyses.

Pearson correlations showed statistically significant associations among study variables. Amotivation correlated with forethought (r = 0.196, *p* < 0.001), self-reflection (r = 0.275, *p* < 0.001), perceived performance control (r = 0.249, *p* < 0.001), and academic engagement (r = 0.278, *p* < 0.001). Forethought and self-reflection were strongly correlated (r = 0.775, *p* < 0.001), and both correlated strongly with perceived performance control (forethought: r = 0.827, *p* < 0.001; self-reflection: r = 0.818, *p* < 0.001). Academic engagement correlated with forethought (r = 0.465, *p* < 0.001), self-reflection (r = 0.508, *p* < 0.001), and perceived performance control (r = 0.527, *p* < 0.001).

To examine the proposed conditional self-regulation framework, a moderated parallel mediation analysis was conducted using PROCESS macro Model 59. In this model, amotivation (X) was specified as the predictor, academic engagement (Y) as the outcome variable, forethought (M1) and self-reflection (M2) as parallel mediators, and perceived performance control (W) as a moderator of both the motivational–regulatory paths and the direct effect of amotivation on academic engagement. This specification allows estimation of stage-1 moderation (X→M), conditional indirect effects through both mediators, and conditional direct effects of X on Y.

### 4.1. Effects of Amotivation and Performance Control on Self-Regulatory Processes

Forethought (M1). The regression model predicting forethought was statistically significant (R^2^ = 0.687, F(3, 526) = 384.26, *p* < 0.001). Amotivation was a significant predictor of forethought (b = 0.134, SE = 0.062, *p* = 0.031), and perceived performance control was also a significant predictor (b = 1.138, SE = 0.089, *p* < 0.001).

Interaction and probing (M1). The amotivation × perceived performance control interaction was statistically significant (b = −0.037, SE = 0.016, *p* = 0.022), indicating that the effect of amotivation on forethought varied across levels of perceived performance control. Johnson–Neyman results and simple slopes at low (−1 SD), mean, and high (+1 SD) perceived performance control are reported in Table 2.

**Figure 2:**
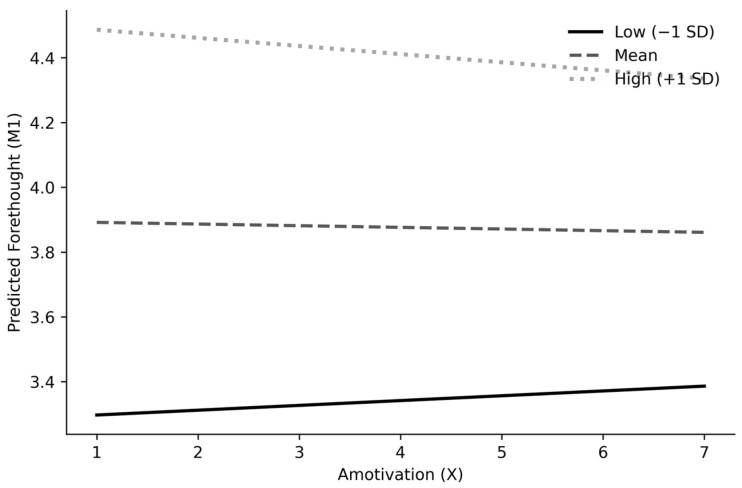
Simple slopes of the amotivation → forethought association at low (−1 SD), mean, and high (+1 SD) levels of perceived performance control (unstandardized predicted values from the stage-1 moderation model).

Follow-up Johnson–Neyman analyses further clarified the nature of this conditional relationship. Specifically, the effect of amotivation on forethought was positive and statistically significant at lower levels of perceived performance control. At lower levels of perceived performance control, the conditional effect was positive and statistically significant; at higher levels, it weakened and became non-significant (Figure 2; Table 2).

Self-reflection (M2). The regression model predicting self-reflection was statistically significant (R^2^ = 0.677, F(3, 526) = 367.33, *p* < 0.001). Amotivation (b = 0.158, SE = 0.073, *p* = 0.031) and perceived performance control (b = 1.225, SE = 0.105, *p* < 0.001) were significant predictors. The amotivation × perceived performance control interaction was not statistically significant (b = −0.031, SE = 0.019, *p* = 0.104).

### 4.2. Prediction of Academic Engagement

Academic engagement (Y). The outcome model was statistically significant (R^2^ = 0.318, F(7, 522) = 34.73, *p* < 0.001). In the full model, the main effects of amotivation (b = 0.182, SE = 0.127, *p* = 0.152), forethought (b = 0.509, SE = 0.518, *p* = 0.326), self-reflection (b = −0.463, SE = 0.465, *p* = 0.319), and perceived performance control (b = 0.324, SE = 0.217, *p* = 0.135) were not statistically significant. Interaction terms were also not statistically significant (amotivation × W: b = −0.027, SE = 0.033, *p* = 0.411; M1 × W: b = −0.123, SE = 0.135, *p* = 0.365; M2 × W: b = 0.176, SE = 0.124, *p* = 0.156). Accordingly, in the outcome equation, there was no statistical evidence for moderation of the amotivation → academic engagement association by perceived performance control (i.e., the X × W interaction was not significant).

Full coefficients are reported in Table 3.

For interpretability of relative predictor contributions, standardized coefficients (β) from a complementary OLS outcome regression without interaction terms are reported in the text (see below), as PROCESS does not output standardized coefficients for moderated models. In that complementary model, perceived performance control showed the largest standardized association with academic engagement (β = 0.302), followed by self-reflection (β = 0.189) and amotivation (β = 0.143), whereas forethought had a small standardized coefficient (β = 0.041). These standardized coefficients are provided solely to describe relative magnitudes and do not replace the unstandardized conditional effects reported by the PROCESS model.

Given the strong intercorrelations among forethought, self-reflection, and perceived performance control (r = 0.775–0.827), we examined collinearity diagnostics. In the outcome regression predicting academic engagement, tolerance values ranged from 0.236 to 0.919 and variance inflation factors ranged from 1.088 to 4.235, indicating moderate multicollinearity, particularly for perceived performance control (VIF = 4.235), forethought (VIF = 3.492), and self-reflection (VIF = 3.403). Collinearity diagnostics also showed elevated condition indices (up to 37.657), suggesting shared variance among predictors. This multicollinearity likely inflated standard errors and reduced the likelihood of statistically significant unique effects in the joint model.

### 4.3. Conditional Direct Effects of Amotivation on Academic Engagement

Conditional direct effects were estimated at low (−1 SD), mean, and high (+1 SD) levels of perceived performance control to describe how the amotivation–engagement association behaves at representative moderator values. Because the X × W interaction in the outcome equation was not statistically significant (Table 3), these conditional estimates should be interpreted as simple conditional effects rather than firm evidence of moderation in the outcome model.

The results indicated that the direct effect of amotivation on academic engagement was statistically significant at all examined levels of perceived performance control, including low (−1 SD), mean, and high (+1 SD) levels. However, the strength of this association was not uniform across control levels. Instead, the magnitude of the effect progressively decreased as perceived performance control increased, indicating a pattern of attenuation rather than elimination.

At lower levels of perceived performance control, amotivation exerted the strongest direct effect on academic engagement (b = 0.095, SE = 0.029, t = 3.33, *p* = 0.001), with the corresponding confidence interval excluding zero. This finding indicates that, when students perceived limited control over their academic performance, higher levels of amotivation were most strongly associated with changes in academic engagement.

At the mean level of perceived performance control, the direct effect of amotivation on academic engagement remained statistically significant (b = 0.081, SE = 0.021, t = 3.91, *p* < 0.001). Although reduced in magnitude relative to the low-control condition, the effect continued to demonstrate a reliable association between motivational disengagement and academic engagement.

At higher levels of perceived performance control, the direct effect of amotivation on academic engagement persisted but was further reduced in strength (b = 0.066, SE = 0.026, t = 2.59, *p* = 0.010). Despite this attenuation, the effect remained statistically significant, indicating that increased perceived control did not fully neutralize the impact of amotivation on academic engagement. The results are presented in Table 4.

Conditional direct effects are reported in Table 4, showing statistically significant effects at low, mean, and high perceived performance control levels.

### 4.4. Conditional Indirect Effects Through Self-Regulatory Processes

Conditional indirect effects were examined to determine whether forethought and self-reflection functioned as mediating mechanisms in the relationship between amotivation and academic engagement under varying levels of perceived performance control. Indirect effects were estimated using a nonparametric bootstrapping procedure with 5000 resamples, and statistical significance was evaluated based on 95% bias-corrected confidence intervals.

The results indicated that the indirect pathway through forethought was not statistically significant at any examined level of perceived performance control. At low levels of control (−1 SD), the estimated indirect effect was small (b = 0.0017) and the corresponding confidence interval included zero (95% CI [−0.0025, 0.0080]). Similarly, at the mean level of perceived performance control, the indirect effect through forethought was near zero (b = −0.0002), with a confidence interval spanning zero (95% CI [−0.0026, 0.0020]). At high levels of perceived performance control (+1 SD), the indirect effect remained non-significant (b = 0.0005; 95% CI [−0.0058, 0.0071]). These findings indicate that forethought did not transmit the effect of amotivation on academic engagement at any level of perceived performance control.

In contrast, a different pattern emerged for self-reflection. At low levels of perceived performance control (−1 SD), the indirect effect of amotivation on academic engagement through self-reflection was positive but not statistically significant (b = 0.0059), as the confidence interval included zero (95% CI [−0.0048, 0.0218]). At the mean level of perceived performance control, however, the indirect effect through self-reflection was statistically significant (b = 0.0081), with a confidence interval that did not include zero (95% CI [0.0017, 0.0187]). This result indicates that, under moderate levels of perceived control, self-reflection functioned as a mediating mechanism linking amotivation to academic engagement.

At high levels of perceived performance control (+1 SD), the indirect effect through self-reflection was again non-significant (b = 0.0071), with the confidence interval narrowly including zero (95% CI [−0.0009, 0.0197]). This pattern suggests that the mediating role of self-reflection was conditional and restricted to a specific range of perceived control. The results are presented in Table 5.

Conditional indirect effects are reported in Table 5. Forethought did not yield statistically significant indirect effects at any moderator level, whereas the self-reflection indirect effect was statistically significant at the mean level of perceived performance control.

## 5. Discussion

The present study examined how amotivation relates to academic engagement during the university transition by testing a conditional self-regulation framework in which motivational, regulatory, and control-related processes are dynamically coupled. The results address the research questions by clarifying (RQ1) the amotivation–engagement link, (RQ2) whether self-regulatory processes transmit this link, (RQ3–RQ4) whether perceived performance control conditions motivational–regulatory pathways, and (RQ5) whether perceived control attenuates the direct association between amotivation and academic engagement. Rather than supporting a classic mediation structure in which self-regulatory processes uniformly transmit motivational effects to engagement, the findings indicate a more differentiated architecture characterized by (a) robust conditional direct effects, (b) selective, stage-specific moderation, and (c) process-specific conditional mediation. This pattern contributes to current debates on motivation, self-regulated learning, and academic adaptation by showing that engagement is shaped not only by which regulatory processes students report, but also by when and under what perceived control conditions these processes become consequential.

This study advances the higher-education motivation literature by modeling academic engagement as the outcome of a conditional self-regulation system rather than a simple direct-effect process. While recent work continues to document engagement as a key marker of university adaptation and persistence [13,14], the present findings extend this line by demonstrating that the motivational–engagement association operates through mechanisms that are both process-specific and context-dependent. In line with contemporary evidence that perceived control is tightly linked to engagement in higher-education learning contexts [31], the conditional process approach clarifies when regulatory processes transmit motivational strain and when they do not, supporting a context-sensitive view of self-regulation in university learning [26,27,28,29]. By providing data from Western Romanian university students, the study also contributes regionally relevant evidence to the predominantly international literature on engagement, self-regulation, and perceived control, with implications for targeted interventions during academic transition.

A central finding addressing RQ1 and RQ5 is that amotivation exerted a statistically significant conditional direct effect on academic engagement across all examined levels of perceived performance control, even after accounting for forethought, self-reflection, and their interactions with control. Although the magnitude of this effect was attenuated as perceived control increased, it remained statistically significant under low, moderate, and high control conditions. This indicates that motivational disengagement constitutes a primary correlate of academic engagement during transition and is not fully offset by self-regulatory processes. The result aligns with theoretical perspectives emphasizing the foundational role of motivation in learning and engagement [44,45,46,47,48,49]. From this standpoint, self-regulation presupposes a minimal level of motivational energy; when motivation collapses, regulatory strategies may be insufficient to sustain engagement. The present findings extend this view by showing that even when students perceive higher control over academic outcomes, amotivation continues to relate to academic engagement—albeit to a lesser degree. In this sense, perceived performance control functions as a damping mechanism rather than a complete buffer, consistent with work that conceptualizes perceived control as a contextual factor related to engagement, while the present outcome model does not provide statistical evidence of moderation via the X × W interaction (Table 3) [48,49].

The results also provide a conditional interpretation of academic adaptation that is consistent with vulnerability-focused perspectives on the transition to university. Specifically, the pattern suggests that self-regulatory processes become consequential only under certain motivational and control configurations, whereas under stronger motivational depletion the direct motivational signal remains prominent. Such a perspective resonates with transition research emphasizing that motivational disruptions may precede and constrain adaptive self-regulation during vulnerability periods [50,51].

Addressing RQ3 and RQ4, one of the most theoretically informative findings concerns the stage-1 moderation of the amotivation–forethought relationship by perceived performance control. The significant interaction indicates that forethought is not uniformly activated in response to motivational strain but operates in a context-dependent manner. Johnson–Neyman probing showed that at lower levels of perceived performance control, higher amotivation was associated with increased forethought, whereas at higher levels of perceived control this association weakened and became non-significant. This positive conditional association should be interpreted cautiously, particularly given the reliance on self-report and the observed collinearity structure. One plausible interpretation is compensatory planning under low control, where students respond to motivational strain by increasing anticipatory regulation. However, alternative explanations are also viable, such as anxiety-driven planning or performance-pressure-related overpreparation, in which planning increases as a reactive coping response rather than as an effective pathway to engagement [52].

This finding also supports theoretical distinctions between context-sensitive regulatory processes and more stable self-system components [39]. Forethought, as operationalized here, appears particularly sensitive to situational appraisals of control, aligning with prior work showing that planning and goal-setting fluctuate in response to perceived efficacy and task demands [48,49,50,51,52,53,54]. Importantly, this helps explain why forethought did not function as a mediator of the amotivation–engagement association in the conditional indirect effects: even when forethought is activated in low-control contexts, such activation does not necessarily translate into engagement once motivational and control signals are jointly considered.

In contrast, and also relevant to RQ3, self-reflection was predicted independently by both amotivation and perceived performance control without evidence of interaction, indicating a comparatively stable motivational–reflective association across control contexts. This pattern is consistent with perspectives conceptualizing reflection as a relatively stable component of self-regulation [55,56]. However, stability does not imply universal effectiveness. Addressing RQ2 and RQ4, conditional mediation analyses revealed that self-reflection transmitted the effect of amotivation to academic engagement only at moderate levels of perceived performance control. Under low control, reflective activity may not translate into adaptive adjustment because perceived efficacy is limited; under high control, reflection may become redundant or less tightly linked to behavioral engagement. This conditional pattern underscores the role of “optimal” control conditions for reflective processes to contribute to adaptation and is consistent with perspectives emphasizing balanced control appraisals in learning contexts [49,57]. It also offers a principled way to reconcile mixed evidence regarding the role of reflection in academic outcomes [56,57,58].

Taken together, the findings support a controlled direct effect architecture rather than a classic mediation model. In this architecture, amotivation shows a persistent direct association with academic engagement, perceived performance control attenuates—but does not eliminate—this association, and self-regulatory processes operate as context-gated mechanisms rather than as primary, uniform transmission channels. This structure is consistent with contemporary conditional process frameworks emphasizing partial, conditional, and moderated mediation [58,59,60]. From a systems perspective, academic adaptation appears to emerge from the dynamic coupling of motivation and control, with self-regulation playing a supportive but bounded role under conditions of motivational strain.

Theoretically, the study contributes to motivation and self-regulation research by demonstrating that academic engagement cannot be fully explained by additive models of motivation and regulation. Instead, engagement reflects a control-gated system in which motivational energy, perceived control, and regulatory processes interact in stage-specific ways. This complements contemporary motivational theorizing by clarifying the boundary conditions under which self-regulatory mechanisms become functional and by highlighting that the persistence of amotivation can remain salient even under more favorable control appraisals [61,62]. The findings also extend self-regulation perspectives by specifying that different regulatory processes (forethought vs. self-reflection) may serve distinct roles and exhibit distinct sensitivities to contextual control conditions [26,27,28,29].

Practically, the findings suggest that interventions focused solely on enhancing self-regulatory skills may have limited impact if amotivation is not addressed directly. Support efforts during transition periods should prioritize restoring motivational meaning and recalibrating perceptions of control, rather than assuming that improved planning or reflection will automatically translate into engagement. Interventions that reduce amotivation and optimize control perceptions—such as autonomy-supportive teaching and attributional retraining—may be especially relevant for transitional student groups [47,48,49,63,64,65]. At the same time, self-regulation training may be most beneficial when applied selectively and context-sensitively: reflective regulation skills appear most likely to contribute to engagement under moderate control conditions, whereas planning-related regulation may reflect compensatory activation under low control conditions rather than a robust pathway to engagement [63,64,65].

From a practical standpoint, these findings suggest that higher education institutions should move beyond one-size-fits-all study-skills interventions and adopt more differentiated control-calibration strategies [31,63,64,65]. In many university settings, students are offered generic workshops focused on planning, time management, or study techniques, yet the present results indicate that the effectiveness of such strategies depends on whether students perceive academic outcomes as at least partially controllable. Accordingly, interventions should not focus exclusively on strengthening self-regulation in the abstract, but also on helping students recalibrate their sense of agency by clarifying which aspects of academic performance can be influenced through effort, strategy adjustment, feedback use, and support-seeking [63,64,65].

The findings also support a more selective approach to regulation training. Because self-reflection emerged as a meaningful mediator only at moderate levels of perceived performance control, reflective assignments, self-evaluation tasks, and metacognitive journaling may be most effective when students already possess a minimally stable sense of academic agency. For students with very low perceived control, reflective activities alone may not be sufficient and may need to be paired with attributional retraining, structured encouragement, and guided success experiences in order to prevent reflection from becoming repetitive self-monitoring without adaptive motivational consequences.

These results are also relevant for digitally mediated higher education. Learning analytics systems, adaptive academic dashboards, or AI-supported tutoring tools could potentially use students’ current motivational-control profiles to tailor feedback more effectively. For example, when low perceived control is detected, such systems could prioritize agency-restoring prompts, concrete action-oriented guidance, and scaffolded planning support rather than assuming that reflective feedback alone will be sufficient. In this way, digital learning environments could become more responsive to the conditional architecture identified in the present study and better prevent amotivation from progressing toward full disengagement.

Several limitations should be acknowledged. First, the cross-sectional design limits causal inference. Although the conditional process model was theoretically grounded and analytically appropriate for testing indirect and moderated pathways [10,59,60], temporal ordering among amotivation, self-regulatory processes, perceived performance control, and academic engagement cannot be definitively established. Longitudinal or experience-sampling designs would better capture the dynamic evolution of these processes during critical transition periods in higher education.

Second, all constructs were assessed using self-report measures, which may be subject to shared method variance and social desirability bias. While self-reports are suitable for capturing subjective motivational and control appraisals [13,33], future studies could integrate behavioral indicators of engagement, learning analytics, or instructor ratings to triangulate self-regulatory activity and academic adaptation. In addition, physiological or cognitive-load measures may help clarify how motivational strain interacts with regulatory effort at the process level [47].

Third, the sample was obtained through convenience sampling and consisted of students enrolled in Romanian higher education institutions. Although the sample size was substantial and heterogeneous in age and educational level, generalizability to other cultural, institutional, or policy contexts remains limited. Future research should test the proposed model across diverse educational systems and instructional climates characterized by different autonomy-supportive or controlling conditions [57,58,59,60,61].

Fourth, while the study differentiated between forethought and self-reflection, other components of self-regulated learning—such as strategy monitoring, effort regulation, and emotion regulation—were not included. Incorporating a broader range of regulatory processes may help determine whether the control-gated pattern observed here extends beyond planning and reflection or whether additional processes function differently under conditions of amotivation [50,54]. Future research should extend the present model by incorporating additional self-regulatory components, including strategy monitoring, effort regulation, and emotion regulation. These constructs are central to contemporary self-regulated learning frameworks and may help explain why some students maintain engagement under motivational strain while others disengage [63,64]. Recent evidence also suggests that self-regulated learning strategies in higher education—particularly in blended and digitally mediated contexts—show differential effectiveness depending on learning demands and contextual conditions, warranting broader process models beyond forethought and self-reflection [65]. In addition, integrating achievement-emotion processes may refine the interpretation of performance control as a contextual factor shaping engagement [66]. Future studies should incorporate additional adaptation indicators (e.g., academic adjustment, persistence intentions, performance/grades, well-being) to test whether the conditional architecture generalizes beyond engagement. Also, the sample was predominantly female (87.5%), which may limit generalizability. Motivational and self-regulatory dynamics can vary by gender, and the observed conditional patterns may reflect gender-skewed processes. Replication with more gender-balanced samples and gender-stratified analyses is recommended.

Finally, perceived performance control was treated as a unitary moderator because it was operationalized as a single composite scale capturing a general appraisal of control over academic outcomes. Future studies could test multidimensional or domain-specific forms of control (e.g., course-level vs. program-level control; task control vs. outcome control) or apply person-centered approaches to examine whether distinct control profiles differentially condition self-regulation and engagement pathways [66]. Despite these limitations, the present study provides a robust foundation for future work by demonstrating that academic engagement, treated here as a key indicator of academic adaptation, is best understood as a control-gated system in which motivation, regulation, and perceived control interact dynamically. Future research building on this framework may further clarify how and when self-regulatory processes can support students facing motivational challenges and inform more targeted, context-sensitive interventions in higher education.

## 6. Conclusions

Overall, the results partially corroborated our hypotheses (H1–H4), indicating that amotivation is a persistent correlate of academic engagement during the university transition and that its effects are best understood within a conditional self-regulation framework. Across low, mean, and high levels of perceived performance control, amotivation showed a statistically significant conditional direct effect on academic engagement, with perceived control attenuating—but not eliminating—this association. This supports a “controlled direct effect” architecture in which motivational depletion remains salient even under more favorable control appraisals.

At the regulatory level, forethought and self-reflection played distinct roles. Forethought showed stage-specific conditionality, consistent with a context-sensitive activation pattern under low perceived control, whereas self-reflection acted as a comparatively stable regulatory process whose mediating function emerged only under moderate levels of perceived performance control. Thus, self-regulation did not operate as a uniform attenuation pattern against amotivation; instead, regulatory mechanisms appeared process-specific and context-gated, clarifying why planning- and reflection-based regulation do not reliably translate into engagement across all control conditions.

The study’s core contribution is to refine models of academic adaptation by demonstrating that engagement cannot be explained by additive relations among motivation, regulation, and control. Rather, academic engagement, treated here as a key indicator of academic adaptation, emerges from their dynamic coupling in which perceived performance control shapes when self-regulatory pathways become consequential. In the present study, academic adaptation was operationalized via academic engagement as a proximal indicator; therefore, conclusions should be confined to engagement-based adaptation rather than broader adaptation domains. Practically, these findings suggest that interventions should prioritize reducing amotivation and optimizing control perceptions, while applying self-regulation training selectively and context-sensitively. Future research should test this model longitudinally and expand it by incorporating additional self-regulatory components (e.g., strategy monitoring, effort regulation, emotion regulation) and multidimensional control appraisals to better capture how adaptation unfolds across time and learning contexts.

## Figures and Tables

**Table 1:** Participant demographics (*n* = 530).

Characteristic	*n*	%
Gender		
Female	464	87.5
Male	66	12.5
Age (years)		
M (SD)	28.86 (9.75)	—
Range	18–56	—
Highest completed education		
High school	218	41.1
Bachelor’s degree	206	38.9
Master’s degree	82	15.5
Post-secondary/vocational (incl. arts & crafts school)	17	3.2
Doctoral degree	4	0.8
Other forms of education	3	0.6

**Table 2:** Regression Results for Stage-1 Moderation Models Predicting Self-Regulatory Processes.

Predictor	Forethought (M1)			Self-Reflection (M2)		
	b	SE	*p*	b	SE	*p*
Constant	−0.382	0.332	0.251	−0.782	0.393	0.048
Amotivation (X)	0.134	0.062	0.031	0.158	0.073	0.031
Performance Control (W)	1.138	0.089	<0.001	1.225	0.105	<0.001
X × W	−0.037	0.016	0.022	−0.031	0.019	0.104
R^2^	0.687			0.677		
F	384.26 ***			367.33 ***		

Note. *n* = 530. Unstandardized coefficients are reported. *** *p* < 0.001.

**Table 3:** Regression results for the final outcome model predicting academic engagement.

Predictor	b	SE	t	*p*
Constant	1.373	0.700	1.96	0.050
Amotivation (X)	0.182	0.127	1.44	0.152
Forethought (M1)	0.509	0.518	0.98	0.326
Self-Reflection (M2)	−0.463	0.465	−1.00	0.319
Performance Control (W)	0.324	0.217	1.50	0.135
X × W	−0.027	0.033	−0.82	0.411
M1 × W	−0.123	0.135	−0.91	0.365
M2 × W	0.176	0.124	1.42	0.156
R^2^	0.318			
F	34.73 ***			

Note. *n* = 530. Unstandardized coefficients are reported. *** *p* < 0.001.

**Table 4:** Conditional direct effects of amotivation on academic engagement at levels of performance control.

Performance Control	Effect (b)	SE	t	*p*	95% CI
Low (−1 SD)	0.095	0.029	3.33	0.001	[0.039, 0.152]
Mean	0.081	0.021	3.91	<0.001	[0.040, 0.121]
High (+1 SD)	0.066	0.026	2.59	0.010	[0.016, 0.117]

Note. *n* = 530. Effects are unstandardized coefficients derived from PROCESS Model 59. Confidence intervals are bias-corrected.

**Table 5:** Conditional indirect effects of amotivation on academic engagement via self-regulatory processes.

**Forethought (M1)**			
**Performance Control**	**Indirect Effect**	**Boot SE**	**95% Boot CI**
Low (−1 SD)	0.0017	0.0027	[−0.0025, 0.0080]
Mean	−0.0002	0.0011	[−0.0026, 0.0020]
High (+1 SD)	0.0005	0.0032	[−0.0058, 0.0071]
**Self-Reflection (M2)**			
**Performance Control**	**Indirect Effect**	**Boot SE**	**95% Boot CI**
Low (−1 SD)	0.0059	0.0067	[−0.0048, 0.0218]
Mean	0.0081	0.0043	[0.0017, 0.0187]
High (+1 SD)	0.0071	0.0054	[−0.0009, 0.0197]

Note. *n* = 530. Bootstrap confidence intervals based on 5000 resamples. Indirect effects are considered statistically significant when confidence intervals do not include zero.

## Data Availability

The original contributions presented in this study are included in the article. Further inquiries can be directed to the corresponding authors.
